# Levels of psychological abuse and their relationship with possible cognitive impairment among rural older adults in China: a cross-sectional study using latent profile analysis

**DOI:** 10.3389/fpsyg.2026.1970971

**Published:** 2026-10-28

**Authors:** Yaxin Liu, Li Pei, Taoyu Jia, Muzi Li, Haoying Dou

**Affiliations:** School of Nursing, Tianjin University of Traditional Chinese Medicine, Tianjin, China

**Keywords:** older adults, possible cognitive impairment, psychological abuse, rural areas, social network

## Abstract

**Aims:**

This study aimed to identify different levels of psychological abuse among rural older adults in China using latent profile analysis and to examine the association between these levels and possible cognitive impairment.

**Methods:**

The study used a convenience sampling method. From July to October 2025, we conducted a cross-sectional study of psychological abuse among rural older adults in Puyang City, Henan Province, China. Participants completed a demographic questionnaire, the Elder Psychological Abuse Scale in Domestic Setting, the Chinese version of Ascertain Dementia (AD8), and the Lubben Social Network Scale-6 (LSNS-6). Latent profile analysis (LPA) was used to identify groups of rural older adults based on dimensions of psychological abuse. Multinomial logistic regression analysis was conducted to explore the association between possible cognitive impairment and psychological abuse group membership.

**Results:**

A total of 502 valid questionnaires were included in the final analysis. Among rural older adults, 88.8% (*n* = 446) were identified as having possible cognitive impairment, and 11.2% (*n* = 56) were not. Three different levels of psychological abuse were identified among rural older adults: low level of psychological abuse group (Group 1, *n* = 207, 41.2%), moderate level of psychological abuse group (Group 2, *n* = 261, 52.0%), and high level of psychological abuse group (Group 3, *n* = 34, 6.8%). Multinomial regression analyses showed that possible cognitive impairment was a significant factor associated with psychological abuse group membership (*p* < 0.05).

**Conclusion:**

Three different levels of psychological abuse were identified among rural older adults, showing significant differences in possible cognitive impairment across group membership. Therefore, future intervention programs should prioritize rural older adults, particularly those with possible cognitive impairment, to implement effective strategies for reducing psychological abuse.

## 1 Introduction

The pace of population aging is accelerating globally. The number of people aged 60 and older worldwide is projected to reach 2.1 billion by 2050, with the proportion of older adults in the population increasing rapidly in low- and middle-income countries (World Health Organization, 2025). In this context, China had 323 million people aged 60 and older by the end of 2025, accounting for 23.0% of its total population (National Bureau of Statistics of China, 2026). Population aging is more pronounced in rural than in urban areas of China (Meng and Jiang, 2018). According to the Fifth Sample Survey on the Living Conditions of Older Persons in Urban and Rural China, approximately 46% of older adults live in rural areas (China Association on Aging, 2024). Compared with urban older adults, rural older adults are more likely to face physical, emotional, and psychological problems (Kigongo et al., 2026). Moreover, due to limited social services, inadequate support services, and remote geographic locations, rural older adults become more vulnerable. These vulnerabilities expose rural older adults to risks such as isolation, psychological disorders, and elder abuse (Zhang et al., 2022). Among these risks, elder abuse is particularly concerning and has lasting consequences, including impaired physical and mental health, reduced quality of life, and even premature death (Askari et al., 2025).

The World Health Organization (WHO) defines abuse as “a single or repeated act, or lack of appropriate action, occurring within any relationship where there is an expectation of trust, which causes harm or distress to an older person” (World Health Organization, 2024). It takes various forms, including psychological abuse, financial abuse, physical abuse, sexual abuse, and neglect. Among these, psychological abuse is the most prevalent form of elder abuse (World Health Organization, 2017). The prevalence of elder abuse across different regions of the world ranges from 14.1% to 27.3%, with the largest proportion being psychological abuse (Kitaw et al., 2025; Yon et al., 2019; Yon et al., 2017). In China, research on psychological abuse is still at an early stage, and relatively few studies have been conducted. Based on the limited available studies, the prevalence of psychological abuse ranges from 5.10% to 15.06% (Li and Nie, 2018; Tao et al., 2020). However, these prevalence rates may be underestimated, as Chinese older adults may be reluctant to report their abuse experiences in order to maintain family harmony and honor (Sun and Yan, 2025). Therefore, there is a need for deeper understanding of psychological abuse affecting rural older adults in China.

To explain the complexity of elder abuse, previous studies have frequently adopted an ecological perspective (Santos et al., 2020). Based on this perspective, Roberto and Teaster (2017) proposed the Contextual Theory of Elder Abuse (CTEA). The theory is built on Bronfenbrenner’s ecological model (Bronfenbrenner, 1979) and the Centers for Disease Control’s socioecological model (Bonnie, 2003). Bronfenbrenner’s ecological model explains human development by focusing on how individuals develop and interact with multiple environments. The CTEA aims to address the complexity of the elder abuse phenomenon from an ecological perspective. It holds that elder abuse is rooted in individual, relational, community, and societal levels. The CTEA primarily emphasizes factors at the individual level (Roberto and Teaster, 2017). At the individual level, elder abuse is understood as resulting from physical, functional, cognitive, and psychological vulnerabilities that create status inequality and power imbalance between older adults and family members. Santos et al. (2020) revealed that cognitive function, social support and chronic diseases were associated with elder abuse. Steinsheim et al. (2022) found that home-dwelling older adults with dementia were subjected to psychological abuse by a spouse in Norway. One possible explanation is that dementia may increase older adults’ need for care and their reliance on caregivers (Sakakibara et al., 2025). Caring for older adults with dementia may adversely affect caregivers’ cognitive and physical health, which may in turn decrease their ability to provide care (Parra-Rizo and Sanchís-Soler, 2022). Lower caregiving competence has been associated with greater caregiver burden (Chan et al., 2018). Steinsheim et al. (2023) found that a high level of caregiver burden was associated with psychological abuse toward older adults, particularly among caregivers of older adults with dementia. Furthermore, within the CTEA framework, economic resources were also considered relevant factors at the individual level. Maleki et al. (2025) reported that lower income was associated with a greater likelihood of psychological abuse among older adults. In addition to individual-level factors, the CTEA also emphasizes the role of relational-level factors in elder abuse.

At the relational level, social engagement is considered a protective factor against elder abuse (Roberto and Teaster, 2017). Donder et al. (2016) found that lower satisfaction with the social network and higher levels of loneliness were associated with greater elder abuse severity. More broadly, social connectedness may also be related to the well-being of older adults. Parra-Rizo and Sanchís-Soler (2023) found that having a diverse social network was positively associated with quality of life among older adults. In another study, older adults who experienced psychological abuse and participated in social activities were reported to be happier than those who did not participate in such activities (Amornkitvikai et al., 2023). Within the CTEA framework, previous studies have also examined relational factors in elder abuse, including co-residence with perpetrators and psychological mistreatment by adult children (Burnes et al., 2025; Lee et al., 2022). Beyond these relational factors, there is a need to examine the association between possible cognitive impairment and elder abuse among rural older adults within the CTEA framework.

Existing research has primarily focused on overall elder abuse among older adults with dementia (Johansson et al., 2024; Kitaw et al., 2025; Shen et al., 2025). Notably, psychological abuse is the most common type of abuse among community-dwelling older adults with dementia, with prevalence estimates ranging from 27.9% to 62.3% (Dong et al., 2014). However, relatively few studies have specifically examined the association between possible cognitive impairment and psychological abuse, particularly among rural older adults. Therefore, the present study examines the association between possible cognitive impairment and psychological abuse among rural older adults in China, addressing an important gap in the existing literature.

Latent profile analysis (LPA) is a person-centered approach that focuses on the heterogeneity of individual responses by classifying individuals into distinct subgroups with similar response profiles (Marsh et al., 2009). Although previous studies have provided important evidence on the prevalence, common forms, and risk factors of psychological abuse, most of them have adopted variable-centered approaches that mainly reflect overall levels of psychological abuse (Wu et al., 2024; Pei et al., 2025). Previous person-centered studies have primarily examined elder abuse broadly, suggesting heterogeneity in elder abuse experiences (Chokkanathan, 2021; Luo et al., 2026; Santos et al., 2019). Few studies have identified latent profiles of rural older adults based on different dimensions of psychological abuse. This present study used the Elder Psychological Abuse Scale in Domestic Setting, which was specifically developed for the Chinese sociocultural context, and assesses psychological abuse across multiple dimensions. Based on these dimensions, LPA was used to explore potential latent subgroups of psychological abuse and to examine the association between possible cognitive impairment and psychological abuse group membership among Chinese older adults in rural settings.

Based on the CTEA and previous studies, this study proposed the following three hypotheses:

*Hypothesis 1*: There are different latent subgroups of psychological abuse among rural older adults in China.

*Hypothesis 2*: Possible cognitive impairment is associated with psychological abuse group membership among rural older adults in China.

*Hypothesis 3*: Social networks are associated with psychological abuse group membership among rural older adults in China.

## 2 Materials and methods

### 2.1 Study design

This study utilized a cross-sectional design to examine the association between possible cognitive impairment and psychological abuse group membership among rural older adults.

### 2.2 Study setting

The study used a convenience sampling method. From July to October 2025, a total of 517 questionnaires were distributed. The study was conducted in 39 villages located in Fukan Township and Qiankou Township, Nanle County, Puyang City, Henan Province, China. Researchers, with the assistance of family and friends, conducted door-to-door visits to recruit eligible participants. Inclusion criteria: aged 60 years and older; basic communication skills and ability to cooperate; willingness and cognitive capacity to provide informed consent. Exclusion criteria: participants with hearing or language impairments; participants who were diagnosed with dementia; participants who refused to cooperate midway.

### 2.3 Data collection

Before the survey, all investigators received unified training to guarantee data consistency. Before completing the questionnaire, all participants received a comprehensive explanation of informed consent. Researchers explained the purpose, content, and significance of this study and emphasized that participation was voluntary and that participants could withdraw at any time. Only participants who fully understood the information and voluntarily signed written informed consent were enrolled. No proxy respondents were used, and no proxy informed consent was obtained. Three researchers worked as a team to conduct face-to-face interviews with the participants. During each interview, the researcher read each questionnaire item aloud, the participant provided their own response to each item, and the researcher recorded the responses. Participants’ rights and privacy were fully protected throughout data collection.

### 2.4 Study size estimation

The required sample size for this study was determined based on the two main analytical methods used: LPA and multinomial logistic regression. For LPA, there is currently no universally accepted method for determining the required sample size. Simulation studies suggest that sample sizes of 300–500 may provide more stable model estimation in LPA (Nylund-Gibson and Choi, 2018). For multinomial logistic regression analysis, G*Power 3.1 was used to approximate the required sample size using linear multiple regression: Fix model, *R*^2^ deviation from zero. With an effect size of *f*^2^ = 0.10, a significance level of 0.05, a statistical power of 0.95, and 14 predictors, the minimum sample size required was determined to be 285. Considering both requirements, the target sample size was set at no fewer than 500 valid participants.

### 2.5 Study materials

#### 2.5.1 Covariates

Covariates were selected based on the CTEA (Roberto and Teaster, 2017) and previous studies. This study included 14 predictors: 12 demographic and health-related covariates, the AD8 score (primary independent variable), and the LSNS-6 score. The 12 demographic and health-related covariates were classified into the individual, relational, and societal levels of the CTEA framework. Individual-level variables included gender, age, education level, monthly household income, chronic disease status, number of chronic diseases, course of disease, and number of medications. Relational-level variables included marital status, number of children, and living arrangement. The societal-level variable was medical insurance coverage.

#### 2.5.2 Elder psychological abuse scale in domestic setting

This is a 16-item measure that evaluates psychologically abusive behaviors within home environments (Liu, 2022). The scale covers four dimensions: disrespect (5 items), isolation (5 items), threat (3 items), and control (3 items). It adopts a 5-point Likert scale ranging from 1 (Never) to 5 (Always). Higher scores signal a higher extent of psychological abuse. The scale had a Cronbach’s *α* of 0.788, a content validity index of 0.994, and a test–retest reliability of 0.954. In this study, the Cronbach’s *α* was 0.740.

#### 2.5.3 The Chinese version of ascertain dementia (AD8)

The Chinese version of AD8 was used in this study (Li et al., 2012). The AD8 is an 8-item tool used to screen for possible cognitive impairment. It assesses several aspects, including judgment, engagement in hobbies and daily activities, repetitive speech, learning ability, time and event memory, financial management, and daily thinking ability. The scale adopts dichotomous yes-or-no responses, with a total score ranging from 0 to 8. A score of 2 or higher suggests possible cognitive impairment. This scale quickly assesses memory impairment and related declines in the ability to perform activities of daily living. Given its brevity and ease of administration, the AD8 can be used for cognitive impairment screening in primary care settings (Karimi et al., 2022). The scale had a Cronbach’s *α* of 0.780 and a test–retest reliability of 0.960. In this study, the Cronbach’s *α* was 0.735.

#### 2.5.4 Lubben Social Network Scale-6 (LSNS-6)

The LSNS-6, originally developed by Lubben et al. (2006), was translated into Chinese by Chang et al. (2018). The LSNS-6 is a brief measure designed to assess social isolation among older adults. The scale consists of two dimensions: family and friend networks. Each item is rated on a 6-point scale from 0 to 5 (0, 1, 2, 3–4, 5–8, ≥9). The total score ranges from 0 to 30, with scores under 12 indicating social isolation. A family or friend network subscale score under 6 suggests family or friend isolation. The Chinese version of the LSNS-6 had a Cronbach’s *α* of 0.832. In this study, the Cronbach’s *α* was 0.869.

### 2.6 Data analysis

Data were analyzed using SPSS 25.0 and Mplus 8.7. Continuous variables were expressed as means and standard deviations (M ± SD). Categorical variables were presented as frequencies and percentages. One-way analysis of variance (ANOVA) and the Chi-square test (*χ*^2^) or Fisher’s exact test were used to identify differences in continuous and categorical variables across the group membership. Potential covariates were selected based on the CTEA and previous studies. The selected covariates were included in the multinomial logistic regression model regardless of their statistical significance in the univariate analysis, with the group membership as the dependent variable. A two-sided *p*-value < 0.05 was regarded as statistically significant.

The four dimensions of the Elder Psychological Abuse Scale in Domestic Setting were used as observed variables, and LPA was conducted using Mplus 8.7 to identify latent subgroups of psychological abuse among rural older adults in China. Starting with a 1-class model and gradually increasing the number of classes, the best-fitting model was determined by evaluating model fit indices: lower values of the Akaike information criterion (AIC), the Bayesian information criterion (BIC), and the adjusted BIC (aBIC) indicated better fit; an entropy value above 0.800 suggested classification accuracy of over 90% (Wen et al., 2023); and *p*-values (*p* < 0.05) for the Lo–Mendell–Rubin test (LMRT) and the bootstrap likelihood ratio test (BLRT) supported that a k-class model fit the data better than the k-1 class model. In addition, class proportions were also evaluated for each model, with profiles containing less than 5% of the sample considered potentially spurious (Ferguson et al., 2019). The criteria only provide a reference for deciding a profile, and the interpretability of each profile was considered in determining the optimal model.

## 3 Results

### 3.1 Characteristics of participants

In this study, a total of 517 questionnaires were distributed. Of these, 15 questionnaires were excluded (9 with missing values, 5 with highly regular response patterns, and 1 with contradictory responses). Consequently, 502 valid questionnaires were included in the final analysis, with a valid response rate of 97.10%. The age of the participants ranged from 60 to 99 years, with a mean age of 73.00 ± 7.05 years (see Table 1).

**Table 1:** Characteristics of participants and their scores on psychological abuse, AD8 and LSNS-6 (*N* = 502).

Variables		*n*	%
Gender	Male	294	58.6
	Female	208	41.4
Age	60 ~ 69 years	140	27.9
	70 ~ 79 years	259	51.6
	80 ~ 89 years	95	18.9
	≥90 years	8	1.6
Education level	Illiterate	280	55.8
	Primary school	88	17.5
	Middle school	107	21.3
	High school or higher	27	5.4
Marital status	Married	431	85.9
	Unmarried/divorced/widowed	71	14.1
Number of children	1 ~ 2	144	28.7
	3	162	32.3
	4	130	25.9
	≥ 5	66	13.1
Living arrangement	Living alone	99	19.7
	Living with spouse	367	73.1
	Living with children	15	3.0
	Living with spouse and children	21	4.2
Monthly household income* (yuan)	≤ 1,000	47	9.4
	1,001 ~ 1,600	107	21.3
	1,601 ~ 2,200	132	26.3
	≥ 2,201	216	43.0
Medical insurance coverage	No	45	9.0
	Yes	457	91.0
Chronic disease status	No	101	20.1
	Yes	401	79.9
Number of chronic diseases	None	101	20.1
	1	243	48.4
	≥ 2	158	31.5
Course of disease	0 years	101	20.1
	1 ~ 9 years	231	46.0
	10 ~ 19 years	124	24.7
	≥ 20 years	46	9.2
Number of medications	None	101	20.1
	1	75	14.9
	2	100	19.9
	≥ 3	226	45.0
Possible cognitive impairment	No	56	11.2
	Yes	446	88.8
Age (M ± SD)	73.00 ± 7.05		
Psychological abuse scores (M ± SD)	28.54 ± 5.38		
AD8 scores (M ± SD)	5.48 ± 2.05		
LSNS-6 scores (M ± SD)	15.13 ± 3.77		

Household monthly income*: own work income, spousal support, government subsidies, and adult children support.

### 3.2 Model selection

Using the scores on the four dimensions of psychological abuse, latent profile models with one to five profiles were sequentially fitted. As shown in Table 2, the entropy values for all models exceeded 0.900, suggesting high classification accuracy. As the number of latent profiles increased, the AIC, BIC and aBIC all showed a gradual decline, suggesting improved model fit based on these indices. However, the LMRT *p*-value for the 4-profile model and both the LMRT and BLRT *p*-values for the 5-profile model were greater than 0.05. In addition, the smallest class proportions in both the 4-profile and 5-profile models were less than 5%, further indicating potentially over-extraction. In terms of classification quality, the entropy of the 3-profile model was 0.943, above the recommended standard of 0.800, indicating good classification accuracy. In addition, both the LMRT (*p* = 0.0034) and BLRT (*p* < 0.001) for the 3-profile model were statistically significant (*p* < 0.05), and all class proportions exceeded 5%. Therefore, the 3-profile model was ultimately selected as the optimal model.

**Table 2:** Fit indices of latent profile model (*N* = 502).

Model	AIC	BIC	aBIC	Entropy	*p*		Class proportions
					LMRT	BLRT	
1-profile	7451.517	7485.266	7459.873	−	−	−	−
2-profile	6960.843	7015.685	6974.422	0.921	<0.0001	<0.0001	0.83328/0.16672
3-profile	6649.111	6725.046	6667.913	0.943	0.0034	<0.0001	0.51992/0.41235/0.06773
4-profile	5941.874	6038.901	5965.898	1.000	0.7808	<0.0001	0.19721/0.66135/0.10558/0.03586
5-profile	5748.577	5866.692	5777.818	0.961	0.0764	0.07930	0.38638/0.27498/0.10558/0.03586/0.19721

AIC, Akaike information criterion; BIC, Bayesian information criterion; aBIC, adjusted Bayesian information criterion; LMRT, Lo–Mendell–Rubin likelihood ratio test; BLRT, bootstrap likelihood ratio test; −, not applicable.

To assess the classification accuracy of the three-profile solution, the average posterior probability for each profile was calculated. The results indicated that the correct classification probability was 98.0% for Profile 1, 98.9% for Profile 2, and 95.5% for Profile 3. All three probabilities exceeded 90%, indicating that good classification accuracy for the three-profile solution (see Table 3).

**Table 3:** Average posterior probabilities for most likely profile membership.

Most likely profile membership	Profile 1	Profile 2	Profile 3
Profile 1	0.980	0.011	0.009
Profile 2	0.011	0.989	0.000
Profile 3	0.045	0.000	0.955

Profile 1: low level of psychological abuse group; Profile 2: moderate level of psychological abuse group; Profile 3: high level of psychological abuse group.

### 3.3 Naming of psychological abuse groups

The total scores of the three identified latent subgroups across the four dimensions are depicted in Figure 1. To facilitate interpretation, scores for each dimension were standardized using the mean and standard deviation of the total sample (mean = 0, standard deviation = 1).

**Figure 1:**
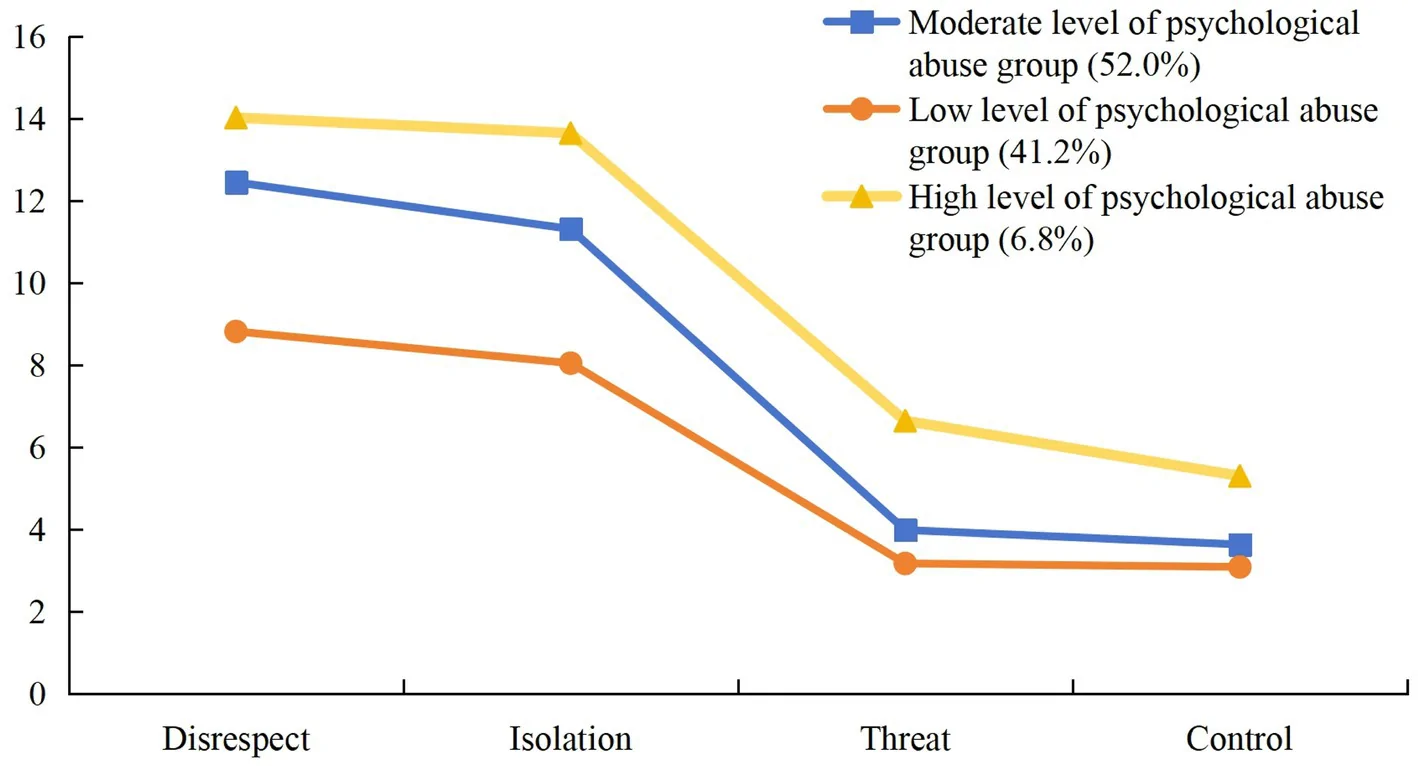
The characteristic distribution of three potential profiles of psychological abuse among rural older adults in China.

#### 3.3.1 Group 1: low level of psychological abuse

The first group comprised 41.2% (*n* = 207) of the sample and was characterized by relatively lower scores on all four dimensions of psychological abuse. All z-score values were lower than mean of the whole sample for each of the dimensions considered. Specifically, low values were observed for the disrespect (*z* = −0.91) and isolation (*z* = −0.93) as well as for the threat (*z* = −0.57) and control (*z* = −0.51) dimensions.

#### 3.3.2 Group 2: moderate level of psychological abuse

The second group was the largest, comprising 52.0% (*n* = 261) of the sample. It was characterized by standardized scores above the sample mean on all four dimensions, with z-scores ranging from 0.13 to 0.56. The standardized scores were relatively higher for the disrespect (*z* = 0.56) and isolation (*z* = 0.53) dimensions, whereas the threat (*z* = 0.14) and control (*z* = 0.13) dimensions were closer to the sample mean.

#### 3.3.3 Group 3: high level of psychological abuse

The third group was the smallest, comprising 6.8% (*n* = 34) of the sample. This group also showed standardized scores above the sample mean on all four dimensions, with particularly high scores for the threat and control dimensions, where z-scores exceeded 2.0. The highest standardized score was observed for the threat (*z* = 2.40) dimension, followed by the control (*z* = 2.09), isolation (*z* = 1.62), and disrespect (*z* = 1.23) dimensions.

### 3.4 Comparison of demographic characteristics in psychological abuse group membership

The univariate analysis showed significant differences among the three groups (*p* < 0.05) in gender, age, number of children, monthly household income, medical insurance coverage, chronic disease status, number of chronic diseases, disease duration, number of medications, AD8 scores, and LSNS-6 scores (see Table 4).

**Table 4:** Univariate analysis of psychological abuse levels (*N* = 502).

Variables		Group 1 (*n* = 207)		Group 2 (*n* = 261)		Group 3 (*n* = 34)		*χ*^2^/*F*	*p*
Gender									
Male		132	(63.8%)	148	(56.7%)	14	(41.2%)	6.918^a^	0.030
Female		75	(36.2%)	113	(43.3%)	20	(58.8%)		
Age									
60 ~ 69 years		83	(40.1%)	49	(18.8%)	8	(23.5%)	31.755^b^	<0.001
70 ~ 79 years		85	(41.1%)	159	(60.9%)	15	(44.1%)		
80 ~ 89 years		35	(16.9%)	50	(19.2%)	10	(29.4%)		
≥ 90 years		4	(1.9%)	3	(1.1%)	1	(2.9%)		
Education level									
Illiterate		105	(50.7%)	152	(58.2%)	23	(67.6%)	5.689^b^	0.446
Primary school		39	(18.8%)	46	(17.6%)	3	(8.8%)		
Middle school		49	(23.7%)	51	(19.5%)	7	(20.6%)		
High school or higher		14	(6.8%)	12	(4.6%)	1	(2.9%)		
Marital status									
Married		183	(88.4%)	222	(85.1%)	26	(76.5%)	3.729^b^	0.166
Unmarried/divorced/									
Widowed		24	(11.6%)	39	(14.9%)	8	(23.5%)		
Number of children									
1 ~ 2		63	(30.4%)	77	(29.5%)	4	(11.8%)	14.894^b^	0.019
3		69	(33.3%)	84	(32.2%)	9	(26.5%)		
4		42	(20.3%)	73	(28.0%)	15	(44.1%)		
≥5		33	(15.9%)	27	(10.3%)	6	(17.6%)		
Living arrangement									
Living alone		41	(19.8%)	49	(18.8%)	9	(26.5%)	4.729^b^	0.544
Living with spouse		156	(75.4%)	189	(72.4%)	22	(64.7%)		
Living with children		4	(1.9%)	10	(3.8%)	1	(2.9%)		
Living with spouse and children		6	(2.9%)	13	(5.0%)	2	(5.9%)		
Monthly household income* (yuan)									
≤1,000		14	(6.8%)	32	(12.3%)	1	(2.9%)	44.626^b^	<0.001
1,001 ~ 1,600		29	(14.0%)	76	(29.1%)	2	(5.9%)		
1,601 ~ 2,200		55	(26.6%)	72	(27.6%)	5	(14.7%)		
≥2,201		109	(52.7%)	81	(31.0%)	26	(76.5%)		
Medical insurance coverage	No	4	(1.9%)	39	(14.9%)	2	(5.9%)	26.795^b^	<0.001	Yes	203	(98.1%)	222	(85.1%)	32	(94.1%)		
	Chronic disease status	No	54	(26.1%)	41	(15.7%)	6	(17.6%)	7.875^a^	0.019	Yes	153	(73.9%)	220	(84.3%)	28	(82.4%)		
Number of chronic diseases									
None		54	(26.1%)	41	(15.7%)	6	(17.6%)	14.489^b^	0.005
1		105	(50.7%)	122	(46.7%)	16	(47.1%)		
≥2		48	(23.2%)	98	(37.5%)	12	(35.3%)		
Course of disease									
0 years		54	(26.1%)	41	(15.7%)	6	(17.6%)	15.182^a^	0.017
1 ~ 9 years		82	(39.6%)	135	(51.7%)	14	(41.2%)		
10 ~ 19 years		57	(27.5%)	59	(22.6%)	8	(23.5%)		
≥20 years		14	(6.8%)	26	(10.0%)	6	(17.6%)		
Number of medications									
None		54	(26.1%)	41	(15.7%)	6	(17.6%)	12.929^a^	0.043
1		29	(14.0%)	41	(15.7%)	5	(14.7%)		
2		47	(22.7%)	48	(18.4%)	5	(14.7%)		
≥3		77	(37.2%)	131	(50.2%)	18	(52.9%)		
AD8		4.14 ± 2.01	6.32 ± 1.44	7.12 ± 1.70	109.223^c^	<0.001			
LSNS-6		16.21 ± 3.77	14.32 ± 3.65	14.71 ± 3.04	15.598^c^	<0.001			

Significance level = 0.05; *χ*^2^ = chi-square test; a = *χ*^2^ test; b = Fisher’s exact test; c = one-way ANOVA test; Group 1: Low level of psychological abuse group; Group 2: Moderate level of psychological abuse group; Group 3: High level of psychological abuse group; Monthly household income*: own work income, spousal support, government subsidies, and adult children support.

### 3.5 Multinomial logistic regression analyses for psychological abuse levels

Multinomial logistic regression analyses were performed with the high level of psychological abuse group serving as the reference group. The dependent variable was the psychological abuse group classification, while independent variables included all selected covariates. Only variables that remained statistically significant in the final model were presented in Table 5. The results showed that possible cognitive impairment was identified as a significant factor associated with psychological abuse group membership (*p* < 0.05). Given the very small size of this reference group (*n* = 34), the resulting estimates should be considered exploratory and may be unstable; therefore, the findings should be interpreted with caution.

**Table 5:** Multinomial logistic regression analysis of groups (*N* = 502).

Model	Variables		*β*	SE	Wald *χ*^2^	*p*-value	OR	95% CI
Group 1 vs Group 3	AD8		−1.236	0.188	43.330	<0.001	0.290	0.201–0.420
	Monthly household income* (yuan) (Ref: ≥ 2,201)	≤1,000	2.416	1.556	2.412	0.120	11.204	0.531–236.390
		1,001 ~ 1,600	1.736	0.871	3.973	0.046	5.674	1.030–31.275
		1,601 ~ 2,200	1.381	0.610	5.121	0.024	3.979	1.203–13.126
Group 2 vs Group 3	AD8		−0.481	0.174	7.656	0.006	0.618	0.440–0.869
	Monthly household income* (yuan) (Ref: ≥ 2,201)	≤1,000	2.746	1.438	3.648	0.056	15.582	0.931–260.933
		1,001 ~ 1,600	2.401	0.825	8.477	0.004	11.034	2.192–55.545
		1,601 ~ 2,200	1.404	0.569	6.081	0.014	4.072	1.334–12.429

OR: odds ratio; CI: Confidence Interval; The reference category is group 3; Significance level = 0.05; Group 1: Low level of psychological abuse group; Group 2: Moderate level of psychological abuse group; Group 3: High level of psychological abuse group; Monthly household income*: own work income, spousal support, government subsidies, and adult children support.

## 4 Discussion

### 4.1 Characteristics of psychological abuse

The mean total score of psychological abuse among rural older adults was 28.54 ± 5.38. This indicated a moderate level of psychological abuse. The score was higher than that reported in previous research (Wu et al., 2024). Several factors may help explain this difference. First, in this study, 79.9% of the participants had chronic diseases, a significantly higher proportion than in previous research (Wu et al., 2024). Similarly, El-Khawaga et al. (2021) reported a 54.9% prevalence of abuse among older adults with chronic diseases. Second, the participants of this study were entirely from rural areas, whereas those in the previous study (Wu et al., 2024) were from both rural (67.1%) and urban (32.9%) areas. Previous studies have also reported high rates of psychological abuse among rural older adults, ranging from 53.8% to 60.2% (Kumar et al., 2024; Okolie et al., 2025). Within the CTEA framework (Roberto and Teaster, 2017), chronic disease status and place of residence are both individual-level factors. Thus, the higher psychological abuse score observed in this study may be related, in part, to the high prevalence of chronic diseases and the rural composition of the sample.

### 4.2 Characteristics of psychological abuse groups

In this study, our hypothesis that rural older adults would show different latent profiles of psychological abuse was partially supported, as the three identified groups differed primarily in overall psychological abuse levels. Notably, all three groups had the highest raw mean scores on the disrespect dimension. This finding is consistent with previous research among rural older women in China, which also reported the highest score for the disrespect dimension (Pei et al., 2025). The relatively high score on the disrespect dimension may be related to the Chinese cultural norm of filial piety (Luo et al., 2026). Chinese older adults often expected their adult children to show respect and provide care in daily life (Liu and Hu, 2021). This may be because some older parents perceive that they made substantial sacrifices for their families earlier in life, and therefore expect respect and reciprocity from their adult children in later life (Tam and Neysmith, 2006). When these expectations are not met, older parents may be more likely to perceive themselves as belittled or disrespected. Therefore, screening and assessment of psychological abuse among rural older adults should assess whether family members respect older adults’ opinions, feelings, and needs during daily interactions and caregiving.

Building on the raw mean results, the standardized scores further illustrated the difference in psychological abuse dimensions across the groups. Specifically, in the low level of psychological abuse group, standardized scores on all four dimensions of psychological abuse were below the sample mean, indicating that this group was mainly characterized by a lower overall level of psychological abuse. Spurk et al. (2020) reported that a group in which all indicators are below the sample mean mainly reflects an overall level difference. This overall low level of psychological abuse group may partly reflect that some rural older adults genuinely experience less psychological abuse. However, the possibility of underreporting should also be considered. In China, the traditional belief that “family problems should be kept within the family” remains common (Luo et al., 2026). Given that psychological abuse often occurs within family relationships, some older adults may be less willing to disclose such experiences. Accordingly, the low scores in this group may reflect both relatively low actual level of psychological abuse and, to some extent, a lower willingness to disclose psychologically abusive behaviors by family members.

In the moderate level of psychological abuse group, the standardized scores for the disrespect and isolation dimensions were relatively high. One possible explanation is that expectations related to filial piety may make older adults more sensitive to disrespectful behavior and infrequent contact from their adult children. For many older adults, adult children are a vital source of care and emotional support. Previous studies have reported that when adult children use rude or abusive language or shout at their parents, older adults may perceive these behaviors as disrespectful (Tam and Neysmith, 2006; Liu and Hu, 2021). In addition, because of work demands, adult children may have less frequent contact with their older parents, which may contribute to feelings of isolation among older parents (Gao et al., 2025). Overall, these factors may help explain the relatively high scores for disrespect and isolation in the moderate level of psychological abuse group.

In the high level of psychological abuse group, it is worth noting that the standardized scores on all four dimensions were above the sample mean. A similar pattern was reported in a study of elder abuse among community-dwelling older adults in China (Luo et al., 2026), which found that the high-abuse group showed high scores on all dimensions. One possible explanation is that as physical functioning declines with age, older adults may become more dependent on their adult children for daily care and financial support (Santos et al., 2020). Adult children often serve as the primary caregivers for their older parents (Wang and Ngok, 2024). Long-term daily caregiving may contribute to caregiver stress and fatigue (Koga et al., 2022). Caregiver stress and fatigue may be associated with a greater likelihood of verbal criticism of older adults and neglect of their needs during daily interactions (Makaroun et al., 2020). Beyond caregiving fatigue, older adults’ dependence on their caregivers may also be associated with greater vulnerability to controlling behavior. Quinlan et al. (2024) suggested that adult children who abuse their parents may take advantage of their parents’ dependence on care and support to undermine their autonomy, for example, by denying their requests, questioning their ability to make decisions, or treating them disrespectfully. Within such controlling relationships, adult children who abuse their parents may threaten to place them in nursing homes (Conrad et al., 2011). In summary, the elevated scores across all four dimensions may be associated with caregiver fatigue and older adults’ dependence on family caregivers.

Overall, the findings of this study are consistent with previous person-centered studies of elder abuse, which have also identified heterogeneity in experiences of elder abuse, although the specific forms of heterogeneity differ across studies (Chokkanathan, 2021; Luo et al., 2026). Chokkanathan (2021) identified three distinct groups of elder mistreatment: high-frequency multiple mistreatment, predominantly psychological mistreatment, and multiple mistreatment predominantly involving neglect, indicating that different forms of mistreatment may be characterized by different combinations. In contrast, this study focused on psychological abuse as a single form of elder abuse. The findings indicate heterogeneity in the severity of psychological abuse among rural older adults, showing that heterogeneity can also exist within a single form of abuse.

### 4.3 Characteristics of possible cognitive impairment

Interestingly, we found a high screening-positive rate for possible cognitive impairment among Chinese older adults in rural areas, with 88.8% of participants aged 60 years and above screening positive using the AD8 tool. Koga et al. (2022) also reported a relatively high rate of 64% among older adults screened for possible cognitive impairment. The rate observed in the present study was higher than those reported in some previous studies. One possible explanation is the use of different cognitive assessment tools, as previous studies often used the CDR, Mini-Cog, MMSE and MoCA (Cai et al., 2026; Ji et al., 2024). For example, an earlier study using CDR tool found cognitive impairment in 61.1% of community older adults aged 60 years and older (Cai et al., 2026). These methodological variations could influence the proportion of older adults identified as having possible cognitive impairment. Meanwhile, differences in the populations studied, including the rural setting of the present study, should also be considered when comparing these findings. In addition, the use of participant-reported AD8 assessments rather than informant-based assessments may affect screening accuracy. Rural older adults with possible cognitive impairment may have difficulty accurately recalling or reporting cognitive changes, which may further contribute to measurement error. In conclusion, these factors may partly explain the high AD8 screening-positive rate observed in this study, although the direction of measurement error cannot be determined.

### 4.4 Influencing factors of psychological abuse group membership

#### 4.4.1 Relationship between psychological abuse group membership and possible cognitive impairment

Interestingly, we found a positive association between possible cognitive impairment and psychological abuse. Specifically, compared with high level of psychological abuse group, the OR for possible cognitive impairment was 0.290 (95% CI: 0.201–0.420, *p* < 0.001) in low level of psychological abuse group, and was 0.618 (95% CI: 0.440–0.869, *p* = 0.006) in moderate level of psychological abuse group. Consistent with our hypothesis, possible cognitive impairment was significantly associated with psychological abuse groups. This finding is consistent with a prior study by Kishimoto et al. (2013), which demonstrated that older adults with mild cognitive impairment were subjected to abuse from caregivers in Japan. From the perspective of the CTEA (Roberto and Teaster, 2017), the association between cognitive impairment and elder abuse may reflect the interplay of multiple contextual factors. Cognitive impairment, including reduced judgment and executive function (McKniff et al., 2026), may impair older adults’ capacity to recognize abuse, avoid potentially harmful situations, or seek help. When these protective abilities are limited, older adults may be more prone to abuse. Additionally, these cognitive changes may lead to greater reliance on caregivers for instrumental activities of daily living (Santos et al., 2020). Such dependency is often accompanied by greater care needs, which may contribute to increased caregiver stress and burden (Reed et al., 2020). Caregivers who experience prolonged caregiving stress and burden without adequate social support may have difficulty coping effectively with their stress (Del-Pino-Casado et al., 2018), which may contribute to physical exhaustion and emotional burnout (Alves et al., 2019). When caregivers become physically exhausted or emotionally burned out, they may have greater difficulty regulating their emotions and behaviors during caregiving interactions, which may lead to verbal criticism or neglect of older adults’ needs (Lee and Kolomer, 2005). The relationship between possible cognitive impairment and psychological abuse may also be bidirectional. In a study of U.S. Chinese older adults, psychological abuse was associated with both lower cognitive functioning at baseline and faster cognitive decline over a two-year period (Li and Dong, 2021). One possible explanation is that prolonged exposure to psychological abuse may contribute to chronic psychological stress among older adults. Prolonged stress may place sustained demands on the body’s stress response system, which may contribute to altered cortisol secretion and regulation (O’Connor et al., 2021). In this context, Lupien et al. (2005) found that persistently elevated cortisol levels are associated with poorer memory performance. Given these findings, future longitudinal studies are needed to examine the potential bidirectional association between older adults with possible cognitive impairment and psychological abuse, and to clarify the underlying mechanisms.

#### 4.4.2 Relationship between psychological abuse group membership and social networks

Contrary to our hypothesis, rural older adults’ social networks were not significantly associated with psychological abuse in this study. This finding was consistent with a prior study by To et al. (2024), which reported that loneliness was significantly associated with elder abuse, whereas social networks were not significantly associated with elder abuse. One possible explanation for the lack of a significant relationship is the way social networks are measured. The LSNS-6 primarily assesses the number of family members and friends with whom older adults have regular contact, can discuss private matters, or can call on for help (Lubben et al., 2006). Although these items capture important aspects of social networks, they may not fully capture relationship quality or perceived social support. In the context of the CTEA (Roberto and Teaster, 2017), it is important to consider not only social networks but also the relationship between the older adults and the perpetrators. Consistent with this perspective, Lee et al. (2022) found that daughters and daughters-in-law were more likely than their male counterparts to engage in psychological abuse of their parents and parents-in-law. This finding suggested that psychological abuse can also occur within close family relationships. In other words, larger social networks do not necessarily indicate that the relationships within that network are supportive or protective. Future research could examine not only social networks but also relationship quality and the relationships between older adults and perpetrators to better understand how these factors are associated with psychological abuse.

#### 4.4.3 Demographic characteristics of psychological abuse group membership

In this study, compared with rural older adults with a monthly household income of ≥2,201 yuan, those with incomes of 1,001–1,600 yuan and 1,601–2,200 yuan had significantly higher odds of belonging to the low or moderate level of psychological abuse groups rather than the high level of psychological abuse group. However, no significant association was observed between a monthly household income of ≤ 1,000 yuan and psychological abuse group among rural older adults. This pattern does not suggest a simple linear relationship between household income and psychological abuse group membership. According to the CTEA (Roberto and Teaster, 2017), elder abuse should be understood by considering health status, living arrangements, and other relevant factors, rather than being explained based solely on economic factors. This study did not further examine the relationships among health status, living arrangements, and economic status among rural older adults. In addition, the observed differences in psychological abuse group across economic levels should be interpreted with caution. Especially some estimates had wide confidence intervals, suggesting limited precision. Future longitudinal studies should examine the relationships of health status, living arrangements, and economic status with psychological abuse group membership.

### 4.5 Clinical and practical implications

This study identified different levels of psychological abuse among rural older adults. These findings may help healthcare professionals tailor interventions to the different levels of psychological abuse. For older adults experiencing low level of psychological abuse, health education may be an important strategy. It may help older adults better understand common forms of psychological abuse, including disrespect, isolation, threat, and control. A better understanding of these behaviors may help them recognize when they experience such behaviors themselves.

For older adults experiencing moderate levels of psychological abuse, interventions should focus on regular follow-up and preventing further worsening of psychological abuse. First, the severity levels of psychological abuse should be monitored regularly. If worsening is observed during follow-up, further assessment should examine the specific forms of abuse and how frequently they occur. Second, in families with high caregiving demands, family members should be encouraged to share caregiving responsibilities and clearly divide daily care tasks, rather than relying on a single caregiver to provide the care (Lim-Soh et al., 2024; Steinsheim et al., 2023). In addition, healthcare professionals may also provide caregivers with practical stress-management guidance, such as taking regular breaks and using simple relaxation techniques.

For older adults experiencing high level of psychological abuse, comprehensive interventions may include psychological assessment and support, legal assistance, and social service support. For psychological assessment and support, professional counselors can assess older adults’ mental health, with particular attention to depressive symptoms (Santos et al., 2020). For those experiencing significant depressive symptoms, counseling may help them express and regulate negative emotions. For legal assistance, legal aid organizations can help older adults understand and safeguard their legal rights. For social service support, social workers may help older adults access available community-based care and support services.

In addition, this study found an association between possible cognitive impairment and psychological abuse among rural older adults. For older adults experiencing psychological abuse, cognitive function screening may also be considered, whereas those whose screening results suggest possible cognitive impairment may also be assessed for psychological abuse. For older adults with both psychological abuse and possible cognitive impairment, healthcare providers may follow them more closely and regularly assess their cognitive function and psychological abuse severity. As psychological abuse often occurs within family relationships, older adults may be reluctant to disclose such experiences. During follow-up visits, healthcare providers should, whenever possible, offer a private setting for communication.

## 5 Limitations

This study has several limitations. First, this study adopted a cross-sectional design. We identified associations between variables but could not establish a causal relationship; longitudinal studies are required to determine causality. Second, the sample had limited geographic and contextual coverage. The sample was geographically restricted to Fukan Township and Qiankou Township, Nanle County, Puyang City, Henan Province, China, which may limit the generalizability of the findings to populations in other regions of China. In addition, this study did not capture community-level factors included in the CTEA, which limited our ability to examine the broader contextual factors related to psychological abuse. Third, the use of convenience sampling may have introduced selection bias, and the use of participant-reported data may have resulted in recall or social desirability bias. Additionally, although the Elder Psychological Abuse Scale in Domestic Setting has been shown to have acceptable reliability among Chinese older adults, lower literacy levels and a higher likelihood of possible cognitive impairment among rural older adults may affect the accuracy of psychological abuse measurement. Fourth, some regression estimates had wide confidence intervals, suggesting limited precision due to small subgroup numbers. In addition, the relatively small number of rural older adults in Group 3 (*n* = 34) may have increased uncertainty in some estimates, as reflected by the wide confidence intervals. Therefore, these estimates should be interpreted with caution. Fifth, possible cognitive impairment was assessed using the AD8 screening tool rather than a formal clinical diagnostic tool. Therefore, the proportion of participants who screened positive should not be interpreted as the prevalence of clinically diagnosed cognitive impairment. Future studies should apply objective multi-dimensional cognitive scales (e.g., MMSE, MoCA, and Mini-Cog). These tools can help further explore the relationship between psychological abuse and possible cognitive impairment.

## 6 Conclusion

This study applied LPA to recognize latent groups of psychological abuse among rural older adults. Three distinct categories were identified: low level of psychological abuse group, moderate level of psychological abuse group, and high level of psychological abuse group. Additionally, our analysis revealed that possible cognitive impairment was a significant factor associated with psychological abuse. In conclusion, future intervention initiatives should target rural older adults, particularly those with possible cognitive impairment. Healthcare providers may tailor intervention strategies to different levels of psychological abuse to better address the needs of older adults, thereby improving the overall well-being and quality of life for older adults.

## Data Availability

The raw data supporting the conclusions of this article will be made available by the authors, without undue reservation.
